# Phylogenomic Analysis of 155 Helminth Species Reveals Widespread Absence of Oxygen Metabolic Capacity

**DOI:** 10.1093/gbe/evad135

**Published:** 2023-07-22

**Authors:** Emma Collington, Briallen Lobb, Nooran Abu Mazen, Andrew C Doxey, D Moira Glerum

**Affiliations:** Department of Biology, University of Waterloo, 200 University Ave W., Waterloo, ON, Canada; Department of Biology, University of Waterloo, 200 University Ave W., Waterloo, ON, Canada; Department of Biology, University of Waterloo, 200 University Ave W., Waterloo, ON, Canada; Department of Biology, University of Waterloo, 200 University Ave W., Waterloo, ON, Canada; Department of Biology, University of Waterloo, 200 University Ave W., Waterloo, ON, Canada; Waterloo Institute for Nanotechnology, University of Waterloo, Waterloo, ON, Canada

**Keywords:** helminths, cytochrome *c* oxidase, peroxisomes, mitochondria, genome reduction

## Abstract

The terminal electron acceptor of most aerobic respiratory chains, cytochrome *c* oxidase (COX), has been highly conserved throughout evolution, from aerobic prokaryotes to complex eukaryotes. Oxygen metabolism in parasitic helminths differs significantly from that of most aerobic eukaryotes, as these organisms can switch between aerobic and anaerobic metabolisms throughout their life cycles. Early studies suggested a lack of COX activity in certain parasitic helminths, and the role of COX in helminth mitochondria remains unclear. To determine whether a functional COX is widely present in helminths, we analyzed the phylogenetic distribution of oxygen metabolism systems across 155 helminth genomes, investigating three distinct sets of protein-coding genes involved in different aspects of oxygen metabolism: COX and its assembly factors, peroxisomes, and the most abundant reactive oxygen species (ROS)-metabolizing proteins. While glycolytic and citric acid cycle enzymes are highly conserved in helminthic species, we observed an apparent widespread absence of essential COX genes across 52% of helminth species investigated. While the most common proteins involved in the defense against ROS are highly maintained across virtually all lineages, we also observed an apparent absence of essential peroxisomal protein-coding genes in 42% of species investigated. Our results suggest that a subset of parasitic helminths utilize oxygen differently from related, nonparasitic species such as *Caenorhabditis elegans*, with significant differences in their mitochondrial electron transport chains and peroxisomes. The identification of substantive differences between parasite and host metabolism offers a new avenue for the development of anthelmintic agents that could target these divergent pathways.

**Significance**

Identifying metabolic differences between parasitic helminths and their hosts is a vital first step in the generation of new anthelmintic agents. Using phylogenomic analysis, we observe the absence of protein-coding genes for critical oxygen metabolic machineries across different helminth lineages. While helminth genomes retain robust antioxidant systems, our results suggest an evolutionary loss of major pathways utilizing oxygen. We find that 52% of species examined appear to have lost genes associated with cytochrome *c* oxidase (the main oxygen metabolizer in mitochondria); 42% appear to lack genes essential for the formation of peroxisomes. Our data suggest that pathways associated with oxygen metabolism in helminths differ substantially from those of other eukaryotes, providing further possible avenues for the development of novel anthelmintics.

## Introduction

Estimated to infect 25% of the human population, parasitic helminths cost global economies billions of dollars through human disability and their impact on livestock and crop industries. Many of the current nematicides used to combat these organisms are toxic to the host and damaging to the environment, while overuse of antiparasitic agents to treat livestock infections has resulted in the development of widespread resistance to these medications (Coghlan et al. 2019). Identifying key differences between parasite and host metabolism is therefore of utmost importance for the development of new therapeutic targets.

Although many features of eukaryotic intermediary metabolism are strongly conserved from the unicellular yeast, *Saccharomyces cerevisiae*, through mammals, a number of deviations have been identified in recent years (Müller et al. 2012; Zimorski et al. 2019), many of which center around the differences between aerobic and anaerobic metabolisms. Five subclassifications of mitochondrial organelles have been described, four of which are ATP-producing and include the classical aerobic mitochondria, which utilize an electron transport chain (ETC) and oxygen as a final electron acceptor. Further mitochondrial variants include organelles that do not utilize oxygen as a final electron acceptor and function anaerobically (Müller et al. 2012). Peroxisomes have been found to have anaerobic and aerobic organelle variants as well: anaerobic peroxisomes that have lost all oxygen-utilizing pathways have been described in anaerobic protists with reduced mitochondria (Le et al. 2020). Despite the existence of these alternate mitochondrial and peroxisomal-derived organelles, certain standard means of oxygen consumption are present in most eukaryotic organisms and an estimated >90% of cellular oxygen is consumed by cytochrome *c* oxidase (COX, Complex IV) of the mitochondrial respiratory chain (Srinivasan and Avadhani 2012).

While an essential metabolite, incomplete reduction of oxygen can result in the formation of reactive oxygen species (ROS) that lead to oxidative damage in cells (Paulsen and Carroll 2013). In addition to mechanisms for dealing with these ROS species, parasitic helminths must also be able to adapt to environments with varying oxygen availability during their distinct lifecycle stages, resulting in a number of unique adaptations in their oxygen metabolizing machinery. Depending on the mode of parasitism, parasitic helminths can use aerobic metabolism, but can also undergo a metabolic shift to anaerobic metabolism for parasitic lifecycle stages where there is limited or no oxygen available (Tielens 1994). In general, adult parasites do not use aerobic oxidation of carbohydrates or oxygen as a final electron acceptor in their ETCs, regardless of oxygen availability (Tielens 1994).

As part of their metabolic adaptation to anaerobiosis, parasitic helminths can reverse succinate dehydrogenase (Complex II of the ETC) to reduce fumarate to succinate (Bueding and Charms 1952). The genes controlling the directional switch in this reaction are differentially expressed throughout development: adult helminths express rhodoquinone, which replaces ubiquinone, for the transport of electrons to Complex II (Tielens 1994). Parasitic helminths have also been proposed to have cytochrome systems that differ from those of their hosts: even under aerobic conditions, their oxygen uptake is lower than would be expected and early studies of *Ascaris lumbricoides* and *Schistosoma mansoni* showed little or no detectable COX activity (Bueding and Charms 1952). COX binds molecular oxygen, which acts as the final electron acceptor in the mitochondrial respiratory chain. The biogenesis of COX is dependent on the actions of a set of highly conserved assembly factors that ensure that the nuclear and mitochondrially encoded subunits, together with the requisite prosthetic groups (heme A and copper), are assembled into a functional holoenzyme (Watson and McStay 2020). The absence of any of these assembly factors, whether providing copper or heme A or chaperoning the mitochondrially encoded subunits, results in the degradation of the catalytic core of the enzyme (Glerum and Tzagoloff 1997). Although there is little research on COX assembly factors in helminths, three assembly factors—Cox11p, Cox17p, and Sco1p (Glerum et al. 1996; Carr et al. 2005; Brière and Tzagoloff 2007)—that are involved in providing copper to the active site subunits are present in *Caenorhabditis elegans* (The UniProt Consortium et al. 2021). Based on their conservation from the unicellular yeast, *S. cerevisiae*, through complex eukaryotes, including *Homo sapiens*, the presence of genes for both the subunits and the assembly factors is essential for the biosynthesis of a functional COX holoenzyme (Watson and McStay 2020).

Besides the mitochondria, peroxisomes are the other major oxygen-consuming organelles, with roles in lipid oxidation and ROS detoxification. Mitochondria and peroxisomes are recognized to have many similarities in their metabolic function and in their ability to adapt in number and morphology to cellular conditions (Schrader and Yoon 2007). In addition to their essential roles in breaking down very long chain fatty acids and scavenging hydrogen peroxide through the activity of catalase, peroxisomes are also required for etherphospholipid biosynthesis, as well as taking part in amino acid, retinol, glutathione, and purine metabolisms (Kanehisa et al. 2021).

Aside from the essential organelles involved in oxygen consumption, eukaryotic cells also contain a conserved set of proteins that are involved in the management of ROS, including superoxide dismutases, glutathione peroxidases, thiolases, peroxidases, thioredoxins, and glutaredoxins. Superoxide dismutases exist in different forms with various metal cofactors (copper-zinc, manganese, iron), and scavenge superoxide radicals, catalyzing their dismutation into molecular oxygen and hydrogen peroxide (Fridovich 1976), which is then dealt with by other antioxidant proteins. Peroxidases are a broad class of enzymes that reduce peroxides using a variety of electron donors, such as glutathione (glutathione peroxidase) (Margis et al. 2008), while other peroxidases rely on redoxins as their electron donors (Arnér and Holmgren 2000). Helminths have both thioredoxins and glutaredoxins and have been documented to have a unique system that links these two redoxins in a multidomain architecture, wherein the thioredoxin and glutaredoxin domains are able to function separately or in concert (Bonilla et al. 2011).

Helminth metabolism has been studied at a biochemical level to only a limited extent, mostly in tractable model systems such as *C. elegans*. Attempts to decipher the mechanisms underlying the observed metabolic alterations in these species using abundant genomic data sets, such as those from the 50 Helminth Genomes Project (Coghlan et al. 2019), have not yet been reported. We therefore chose to investigate the phylogenomic distribution of protein-coding genes associated with three specific aspects of eukaryotic oxygen metabolism, which were not described in the Parasitic Helminth Consortium's enzymatic pathway analysis of the 50 Helminth Genomes Project: 1) COX, as the terminal oxidase in mitochondria, 2) oxidative functions of peroxisomes, and 3) the most abundant cellular antioxidant defense machineries. In this work, we identify previously undescribed alterations to major oxygen metabolic machineries as encoded in the genomes of 155 helminth species, including the widespread absence of key subunits of COX and related assembly factors and the complete absence of peroxisomes in many parasitic species. This large-scale metabolic restructuring of helminths provides a unique opportunity for the development of new anthelmintic agents that specifically target the parasitic metabolism, thereby reducing the toxicity to the host.

## Results

### Construction of Genome-based Phylogenetic Tree

**Fig. 1.**
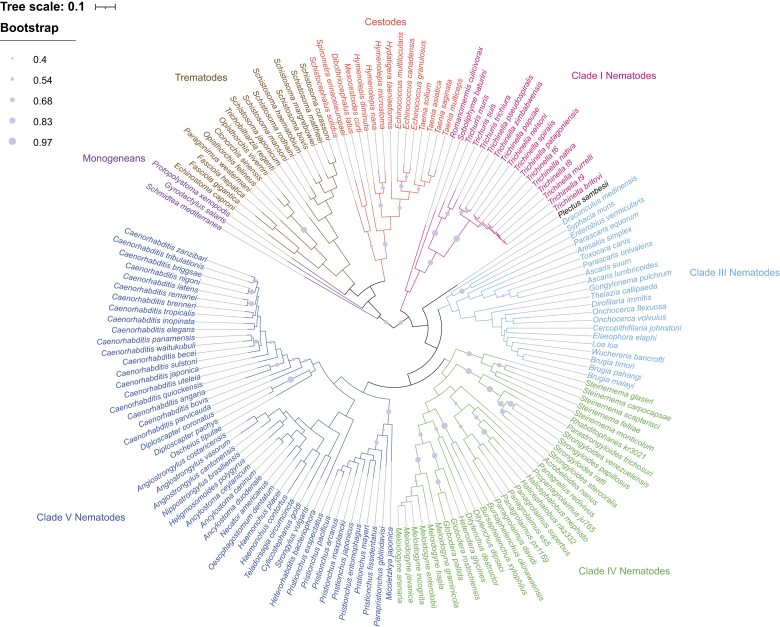
Phylogenetic tree of 155 species of helminth. This tree is based on 339 orthogroups identified with OrthoFinder. Major taxonomic classifications are color-coded, with the exception of *Plectus sambesii,* which is shown in black as it does not cluster with any specific clade. Phylogenetic tree visualization was done using iTOL. Bootstrap values between 0.4 and 1 are shown on the branches.

The observation that parasitic helminths lacked COX activity was initially reported by Bueding and Charms in 1952 and was assumed to be associated with the switch to anaerobiosis. However, given the many metabolic variations in helminths that have now been described, including anaerobic respiration and aerobic glycolysis (Müller et al. 2012), we wondered whether the availability of genomic information from WormBase ParaSite (Howe et al. 2017) might be exploited to shed more light on the initial observations made decades ago. To assess the patterns of presence and absence for protein-coding genes of interest, we began by constructing a genome-based phylogenetic tree of 155 helminth species (fig. 1), based on highly conserved single-copy protein families identified in our data set, using OrthoFinder (Emms and Kelly 2019, see Materials and Methods). The tree includes species from the two major classifications of helminth worms: platyhelminths (monogeneans, cestodes, and trematodes); and clades I, III, IV, and V nematodes (fig. 1). No genomic sequence data is currently available for free-living clade II nematodes and as such they are entirely absent from this tree. Species in our tree cluster according to their taxonomy (i.e., nematode clade or platyhelminth class) and are in excellent agreement with the phylogenetic analysis generated by the International Helminth Genomes Consortium (Coghlan et al. 2019).

### Phylogenomic Distribution of Mitochondrial Aerobic Metabolism

Based on our interest in better understanding COX assembly in health and disease, we first asked whether genes encoding the COX subunits and assembly factors were present in our species of interest. Because COX is the aerobic endpoint of oxidative metabolism, we started our investigation by looking at the overall process of cellular respiration. We examined each of the 155 species for genes involved in glycolysis, the citric acid cycle, and core subunits essential for each of complexes I–III of the mitochondrial ETC (see supplementary fig. S1, Supplementary Material online). With few exceptions, genes encoding all the major glycolytic and citric acid cycle proteins were present across all our species of interest (see supplementary fig. S1, Supplementary Material online). We did initially observe an absence of phosphoglycerate mutase (PGM) in many of the species of platyhelminth, but this could be attributed to the use of a *C. elegans* protein as our query sequence for searches. *Caenorhabditis elegans* and related free-living nematodes possess only an independent PGM (iPGM) protein that operates without cofactors (Dhamodharan et al. 2012). Of our 155 species, 119 had hits for the iPGM query protein from *C. elegans*. For the remaining 36 species, in which we observed an absence of iPGM, we theorized that they must instead have a cofactor-dependent PGM enzyme (dPGM). To verify this, we carried out a BLASTP search using the dPGM protein from *S. cerevisiae* as a query. This resulted in hits for 35 of the species lacking the iPGM, although many species with an iPGM also had hits for the dPGM query. For the single remaining species (*Schistocephalus solidus*), which had hits for neither the iPGM nor the dPGM, we were able to find a PGM protein using the WormBase ParaSite annotations.

**Fig. 2.**
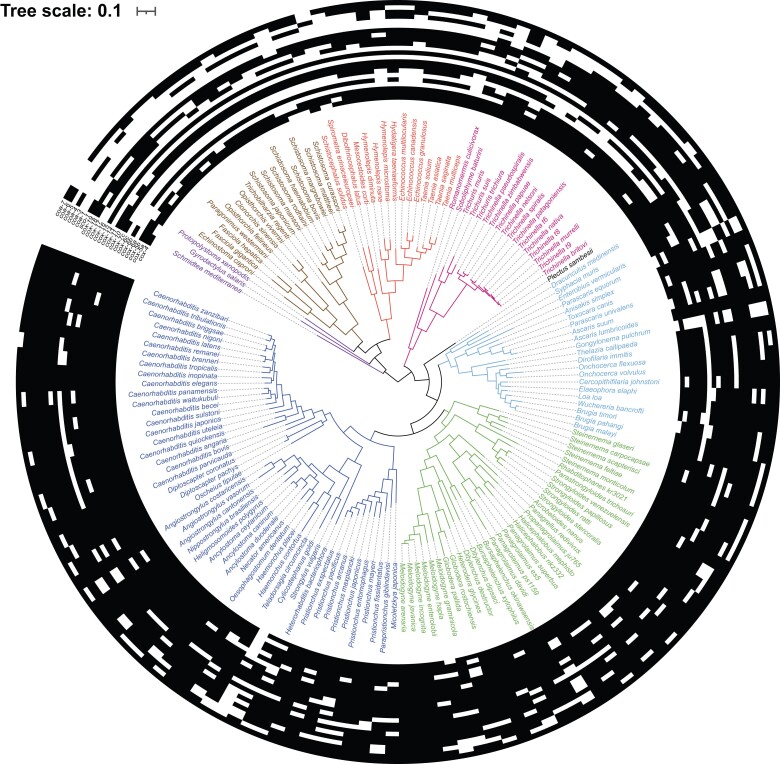
Presence/absence of major nuclear-encoded COX subunits and assembly factors across the helminth phylogenetic tree containing 155 species. The detected presence (black) and absence (white) of the genes encoding the following proteins were mapped onto the helminth phylogenetic tree (from inside to outside): subunits *cox-4*, *cox-5a*, *cox5b*, *cox-6a*, *cox-6b*, *cox-6c*, and *cox-7c*; and assembly factors *cox-10, cox-11*, *cox-14*, *cox-15*, *cox-16*, *cox-17*, *cox-18*, *cox-19*, *sco1, coa-1*, *coa-3*, *coa-4*, *coa-5*, *coa-6*, and *coa-7*. For the percentage of genes of interest present in each species, see supplementary figure S6, Supplementary Material online.

Similar to our results for glycolysis and the citric acid cycle, marker genes for Complex I (*nduf-2.2*), Complex II (*sdh-1*), and Complex III (cytochrome *c*_1_) of the ETC were universally present (see supplementary fig. S1, Supplementary Material online). For COX, *cox-*1, along with the other mitochondrially encoded subunits, *cox-*2, *and cox-*3, encode the catalytic core of COX and are essential to COX function (Okimoto et al. 1992) and are thus usually used as markers for the complex. However, we were unable to obtain estimates of mitochondrial genome coverage for genomic data available in the WormBase ParaSite database. In addition, there are known technical difficulties associated with sequencing helminth mitochondrial DNA (mtDNA), which include a lack of high-quality reference sequences, small mitochondrial genome size, and the AT-rich nature of helminth mtDNA (Jia et al. 2012). Therefore, in order to assess the presence of COX in the species represented on our tree, we identified the core set of nuclear-encoded COX proteins (subunits and assembly factors) present in *C. elegans* using the Universal Protein Knowledgebase (The UniProt Consortium et al. 2021). *Caenorhabditis elegans* COX has peripheral subunits encoded by the nuclear genes *cox-4*, *cox-5a, cox5b*, *cox-6a*, *cox-6b*, *cox-6c*, and *cox-7c*. We additionally identified the presence of a set of assembly factors: *coa-1*, *coa3-7*, *cox 10-11*, *cox14-19*, and *sco-1*, which are homologs of the well-characterized yeast and human proteins. We performed BLASTP searches for the entire complement of nuclear-encoded protein subunits and assembly factors and observed that a number of genes encoding COX proteins appear to be absent in many species from different lineages, including all three classes of platyhelminths, most clade I nematodes, many clade III nematodes, and some clade IV and clade V nematodes (fig. 2). The only species found to encode all COX subunits and the associated assembly factors were clade V nematodes, especially members of the *Caenorhabditis* genus (including *C. elegans*), with other clade IV nematodes also having some members with all subunits and assembly factors.

To eliminate the possibility of other helminth species having additional (previously unidentified) subunits and assembly factors, we searched our annotated proteomes for proteins associated with COX assembly, but did not find any aside from those in *C. elegans* listed above. Overall, the platyhelminths and clade I nematodes have fewer of the nuclear encoded subunits and assembly factors, and most species of these lineages are lacking *cox-17,* without which COX cannot be assembled (Glerum et al. 1996).

Based on the well-understood roles of nuclear-encoded COX assembly factors *cox-11*, *cox-17,* and *sco-1*, which are essential for assembly, the presence of these proteins are predictive of a functional COX holoenzyme. Only 48% of our species of interest were found to have all three of the genes encoding these essential assembly factors, further suggesting that COX is absent from many species of helminth (see supplementary fig. S2*A*  and  *B*, Supplementary Material online).

While the apparent absence of genes from these data sets could potentially be attributed to incomplete genomic data, we noticed that genes related to COX and its assembly were absent at a higher frequency than other genes we examined. In the case of incomplete genomes, we would expect to see a similar distribution of genes absent across all pathways investigated, and our analysis of genes related to glycolysis and the citric acid cycle, processes which are highly conserved in eukaryotes, allowed us to establish a baseline of gene absence/presence for a metabolic process. The absence of genes related to COX is demonstrably higher than the baseline set by the other cellular metabolic processes we interrogated (see fig. 4). Overall, 97% of genes related to glycolysis were present in our species, 99% of TCA cycle genes were present, and for the ETC, marker proteins for Complexes I through III were present in 100% of species. However, three essential assembly factors for COX were only present in 48% of species. Our findings agree with the metabolic studies done in *S. mansoni* (Bueding and Charms 1952), where little or no COX activity was observed. In our data set, *S. mansoni* only had 59% of the COX-related protein-coding genes investigated. The absence of the genes for both subunits and the requisite assembly factors thus predicts a complete absence of COX in a large subset of helminths, especially the parasitic platyhelminths.

### Widespread Absence of Peroxisomal Marker Genes in Platyhelminths and Some Nematodes

**Fig. 3.**
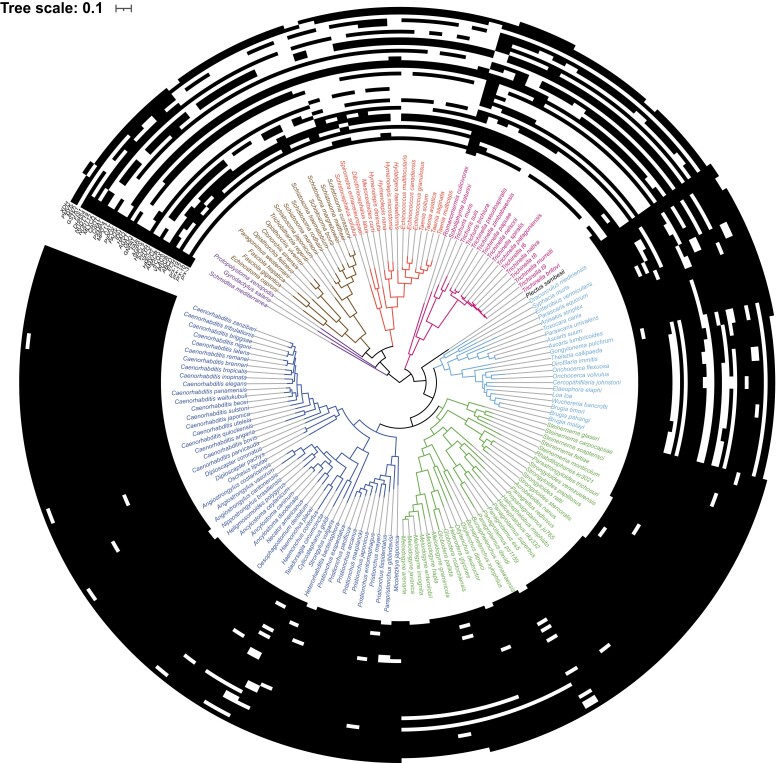
Widespread absence of peroxisomal protein-coding genes across the helminth phylogeny. Detected presence (black) and absence (white) of the following protein-coding genes were mapped onto the tree (from inside to outside): *prx-3*, *prx-10*, *prx-12*, *prx-19*, ECH, ABCD, PDCR, VLACS, ACOX, AMACR, HMGCL, SPCX, PHYH, HPCL2, AGPS, FAR, GNPAT, DDO, AGXT, HAO, IDH, PAOX, CAT, MPV17, PXMP4, CRAT, CROT, MLYCD, NUDT12, NUDT19, DHRS4, FIS1, GSTK1, MVK, PMVK, and XDH. For the percentage of peroxisomal genes of interest, see supplementary figure S7, Supplementary Material online. For a full list of nonabbreviated proteins and the associated peroxisomal pathway, see supplementary table S2, Supplementary Material online.

Because we found such a striking absence of the terminal oxidase for the mitochondrial respiratory chain, we asked whether peroxisomes would be similarly impacted, and in similar lineages. As shown in figure 3, there appears to be a widespread absence of peroxisomal genes in the species studied here. Similar to the results for the COX-associated genes, *C. elegans* and other members of the *Caenorhabditis* genus, as well as free-living clade V nematodes, encode the most complete set of peroxisomal proteins. The platyhelminth and clade I nematodes, however, lack many peroxisomal genes—not just those associated with antioxidant function, such as catalase, but also those related to other peroxisomal metabolic pathways. As a definitive marker for the presence of peroxisomes, we looked specifically for four peroxin-coding genes—*prx-3*, *prx-10*, *prx-12*, and *prx-19*, which encode a core set of peroxisomal biogenesis proteins that are conserved in all eukaryotic lineages (Jansen et al. 2020). These peroxins are essential for the biogenesis of the organelle itself: *prx-3* and *prx-19* are necessary for the sorting of peroxisomal membrane proteins, while *prx-10* and *prx-12* function in receptor recycling and ubiquitination (Jansen et al. 2020). Only 58% of species were found to encode all four of these peroxins, strongly suggesting that the entire organelle could be absent from the remaining 42% of these organisms (see supplementary fig. S3*A*  and  *B*, Supplementary Material online). As with the absence of COX assembly factors, we identified species that had different combinations of these four essential peroxins, with clades III and V nematodes having the highest rates of all four present together.

**Fig. 4.**
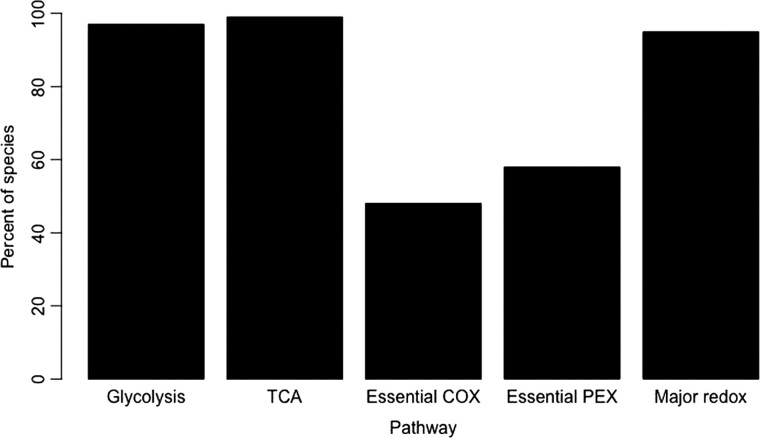
Percentage of helminth species of interest found to possess genes from pathways of interest. 96% of species investigated contained all the genes encoding enzymes essential for glycolysis, and 99% had all genes for the TCA cycle. Only 45% of species contained all three of the essential COX assembly factors *cox-11*, *cox-17*, and *sco1*; 55% of these species had lost the gene for at least one of these. Similarly, only 54% of species had all four peroxin proteins required for the formation of peroxisomes (*prx-3, prx-10, prx-12,* and *p4x-*19); 46% of species did not contain the gene for at least one of these essential peroxisomal biogenesis proteins. Major antioxidant proteins were highly conserved, with 95% of species containing at least one member of each of the following antioxidant protein families: Sod_Cu, Sod_Fe, glutathione peroxidase, thiolase, peroxidase, thioredoxin, and glutaredoxin.

In agreement with a previous study (Žárský and Tachezy 2015), the platyhelminth lineages had a marked number of absences for genes encoding peroxisomal proteins (more than 50% in many cases see supplementary fig. S3*A*  and  *B*, Supplementary Material online), including catalase. As a peroxisomal organelle is unlikely to be present given this number of apparent absences, the remaining genes could be functioning in other pathways, perhaps within the mitochondria. For example, enoyl CoA hydratase is a beta oxidation protein that can also be present in the mitochondria (Kanehisa et al. 2021), which would account for its presence in all the species investigated. We also observed an absence of many peroxisomal genes in clade I and clade III nematodes in our data set, with absences in some parasitic clades IV and V nematodes as well, although to a lesser extent than the other lineages.

Indeed, a complete loss of the peroxisome in nine species of nematode and all platyhelminths has been proposed (Žárský and Tachezy 2015). Other nonhelminth anaerobic eukaryotes have been observed to lose their peroxisomes, or to contain so called “anaerobic peroxisomes’, which lose their oxygen-utilizing metabolic pathways while retaining other peroxisomal functions (Le et al. 2020). The absence of peroxisomal genes, including the essential peroxins, we observed was more extensive and occurred in more lineages of helminth than previously proposed.

Interestingly, an overlay of the presence/absence profiles generated for the COX-related genes and the peroxisomal genes (see supplementary fig. S4, Supplementary Material online) reveals that many of the parasitic species that we predict to lack a functional COX are also predicted to lack peroxisomes. Our analysis shows that 21% of the species interrogated here lack both the terminus of the canonical eukaryotic mitochondrial respiratory chain and the typical suite of peroxins considered essential in higher eukaryotes (supplementary fig. S4, Supplementary Material online). Only 26% of the species on our phylogenetic tree had all the essential marker genes for both COX and peroxisomes. A further 21% had absences of at least one essential peroxin gene, while retaining the essential COX assembly factors (see supplementary table S1, Supplementary Material online for a complete list of species with each set of protein-coding genes present/absent).

### Broad Conservation of Genes Encoding Antioxidant Proteins in Helminth Genomes

Given the absence of two classical sources of oxygen consumption in eukaryotes, we sought to determine whether there would be any consequences for proteins associated with cellular antioxidant defenses. We identified genes encoding superoxide dismutase in helminths with copper (Sod_Cu) and iron (Sod_Fe) cofactors, as well as genes encoding other redox proteins (glutathione peroxidase, thiolase, peroxidase, thioredoxin, and glutaredoxin) present in our species of interest, using PfamScan to annotate predicted proteomes. Although catalase is involved in defense against ROS, we chose to include it as a peroxisomal protein-coding gene and so it does not appear in this data set. Unlike the peroxisomal proteins and COX subunits and assembly factors, the antioxidant protein-coding genes we searched for, which represent a broad swath of cellular antioxidant defenses, are widely present in the helminth species investigated here, with 95% of species having all the antioxidant proteins investigated (see supplementary fig. S5, Supplementary Material online).

As part of oxygen-related metabolic capacity, parasitic helminths must have robust antioxidant defense systems to cope with bursts of ROS released by their hosts (Tielens 1994). If peroxisomes, along with catalase, are lost, other antioxidant proteins will be of increasing importance to the survival of these organisms. Not surprisingly, therefore, we found almost universal retention of the antioxidant proteins interrogated across our species of interest. We additionally identified hundreds of protein domain architectures (including domains associated with antioxidant proteins) that are entirely unique to helminths (data not shown), which is consistent with previous observations of large numbers of novel domain combinations encoded in parasitic helminth genomes (Coghlan et al. 2019). The functional roles for these novel architectures remain unknown but could relate to enhancements in antioxidant defenses.

## Discussion

Helminths are known to be associated with rapid evolution, with each parasitic species having undergone specific genomic adaptations that allows it to exist within its host niche. Parasitic flatworms have undergone extensive genome reduction relative to their nematode counterparts, resulting in the absence of many metabolic pathways, especially those related to digestion and auxiliary metabolism (Zarowiecki and Berriman 2015). The existence of a different cytochrome system associated with mitochondrial respiration has also been suggested (Bueding and Charms 1952). Certain species of helminth (*Parascaris univalens*, *Ascaris suum*, and *Strongyloides papillosus*) have also been observed to undergo chromosomal diminution between lifecycle stages, resulting in the elimination of many protein-coding genes in all but the germline cells (Wang and Davis 2014). Our analyses of worm genome data have revealed a much broader loss of coding capacity for two major consumers of molecular oxygen in higher eukaryotes, namely COX and peroxisomes.

The apparent widespread absence of cytochrome oxidase suggests that these helminths must utilize an alternative electron acceptor in their mitochondrial electron transport chains, although we were only able to identify the presence of the canonical alternative oxidase (McDonald and Gospodaryov 2019) in two clade IV species, *Ditylenchus destructor* and *Ditylenchus dipsaci*. It is likely that most of these species undergo fermentation involving malate dismutation, a common pathway identified in a variety of parasitic helminths. It is also possible that some of these species could use fumarate as an electron acceptor, which is the most common form of anaerobic respiration described in eukaryotes (Müller et al. 2012). Most of the species investigated here do contain the genes, as recently identified in *C. elegans* (*kynu-1* and *coq-2*) (Del Borrello et al. 2019), needed for the biosynthesis of rhodoquinone (data not shown), which is the obligate electron carrier in the reduction of fumarate to succinate by fumarate reductase. However, the absence of the genes encoding COX subunits and assembly factors precludes a role for typical mitochondrial aerobic metabolism that has been presumed to function in the free-living and early larval stages of these species (Müller et al. 2012). It has long been thought that the lack of COX in the adult platyhelminths would be due to a down-regulation of oxidative phosphorylation capacity, perhaps in a manner similar to the aerobic metabolic suppression observed in hibernating animals (Mathers and Staples 2019). Because the helminths are able to utilize anaerobic metabolism, some of the mitochondrial proteins involved in aerobic respiration may have become redundant and the genes therefore lost. The apparent loss of COX-encoding genes we identify here also differs from the loss of ferrochelatase, an essential enzyme for heme biosynthesis, documented by the Helminth Genome Consortium, which noted the presence of ferrochelatase-like genes of unknown function (Coghlan et al. 2019). Because the family of cytochrome *c* oxidases is so strictly conserved, based on its characteristic prosthetic groups and cofactors, any COX-like genes should be readily identifiable and are unlikely to have undergone sequence divergence to the point that they are no longer detected, although we cannot rule out this possibility. It is also possible that different species of helminth require a different set of COX assembly factors than those identified in *C. elegans*, though this seems unlikely given that *cox-17*, *cox-11*, and *sco-1* are highly conserved and required for the formation of COX in almost all other eukaryotes studied (Watson and McStay 2020). Interestingly, we note that many species had one or more of these three assembly factors present even in the absence of nuclear-encoded COX subunits, often within multidomain protein architectures (data not shown), which might suggest additional cellular roles for these proteins. The overlapping absences of COX-associated and peroxisomal genes further suggests that peroxisomal loss in the reductive genome evolution of anaerobic eukaryotes, such as helminths, may be followed by the loss of genes encoding mitochondrial proteins involved in aerobic metabolism. Although the absence of peroxisomes has been previously documented in platyhelminths, we were still able to identify approximately half of our peroxisomal query proteins in these species, and *prx-10*, one of the essential peroxins was found in 95% of species on our tree, even in those species known to be missing peroxisomes. This suggests that the retained peroxisomal biogenesis genes, including *prx-10*, might also be performing other roles within the cell. This is similar to what we see in the COX data, which retain some COX-related assembly proteins in species that cannot assemble the enzyme's catalytic core. Ongoing studies are aimed at determining whether these assembly factors also have roles outside of COX assembly.

The absence of protein coding genes we have uncovered here could be due to either evolutionary reductive genome evolution or technical problems related to partial genome completeness. Variations in genome completeness are unlikely to account for the significant number of absent genes of interest here, as seen for the members of *Trichinella* in the clade I nematodes, which have complete BUSCO values comparable to those of *Caenorhabditis* species in clade V (see supplementary fig. S8, Supplementary Material online). We were only able to detect 46% of possible peroxisomal genes in *Trichinella* lineages (see supplementary fig. S7, Supplementary Material online), and ten of the 12 species of *Trichinella* had absences of at least one essential COX assembly factor (either *cox-11* or *cox-17*, see supplementary fig. S2*A*, Supplementary Material online), although these genes are highly retained in the clade V nematodes. The possibility that some species are able to undergo chromatin diminution could also result in the gene absences observed, although this phenomenon has only been documented in certain species of clade III nematode (*A. suum*, *A. lumbricoides*, *Toxocara canis*, and *P. univalens*) (Tobler et al. 1992). This process, which could only account for absences in these few species, thus seems unlikely to explain the widespread absences observed here. As is always the case for gene-based predictions, it is difficult to ascertain whether the identified protein-coding genes encode expressed, functional proteins, especially given the possibility of pseudogenes and the presence of multidomain fusion architectures whose functions are currently unknown.

Assuming that the absent genes identified here are the result of reductive genome evolution, our analyses suggest that a wide array of helminths, especially the parasitic worms, have lost their peroxisomes, along with the canonical mitochondrial terminal oxidase. Understanding the alternate metabolic pathways being used in the platyhelminths could identify potential new targets for future anthelmintics. Rhodoquinone, essential for malate dismutation via fumarate reductase, is a potential target as it is not found in hosts, and a high throughput screening assay has recently been developed to investigate compounds that inhibit rhodoquinone (Del Borrello et al. 2019). Another candidate target might be a cytosolic oxygen-consuming NADH oxidase, similar to those found in a number of parasitic excavates and amoebae, which could support anaerobic respiration (Müller et al. 2012). Our analyses reveal a loss of oxidative metabolic capacity that suggests there are further unique metabolic adaptations in worms that could provide avenues for experimental investigation toward the generation of new anthelmintics.

## Materials and Methods

### Domain-based Functional Annotation of WormBase ParaSite Proteomes and Phylogenetic Analysis

We utilized genomic data and gene predictions for 155 species of nematode and platyhelminth that were available on WormBase ParaSite as of September 2022 (Howe et al. 2017), including sequence data from the 50 Helminth Genomes Project (Coghlan et al. 2019) and other previously published helminth genomes (Bai et al. 2013; Bennett et al. 2014; Bernard et al. 2017; Blanc-Mathieu et al. 2017; Cotton et al. 2014; Desjardins et al. 2013; Dillan et al. 2015; Reshod et al. 2019; Eves-van den Aaker et al. 2016; First et al. 2015; Forth et al. 2014; Fradin et al. 2017; Ghedin et al. 2007; Godel et al. 2012; Grohme et al. 2018; Hahn et al. 2014; Hiraki et al. 2017; Hunt et al. 2016; Jex et al. 2014; Kanzaki et al. 2018; Kikuchi et al. 2011; Korhonen et al. 2016; Li et al. 2018; Luo et al. 2019; Maldonado et al. 2017; Masonbrink et al. 2019; Mimee et al. 2019; Mitreva et al. 2011; Mortazavi et al. 2010; Nemetschke et al. 2010; Nowak et al. 2020; Oey, Zakrzewski, Gravermann, et al. 2019a; Oey, Zakrzewski, Narain, et al. 2019b; Opperman et al. 2008; Prabh et al. 2018; Protasio et al. 2012; Ragsdale et al. 2019; Rödelsperger et al. 2017; Rošić et al. 2018; Schiffer et al. 2013; Schiffer et al. 2018; Schiffer, Danchin, et al. 2019; Schwarz et al. 2015, 2013; Serra et al. 2019; Small et al. 2016; Somvanshi et al. 2018; Srinivasan and Avadhani 2012; Srinivasan et al. 2013; Stevens et al. 2019; Stroehlein et al. 2019; Sun et al. 2020; Szitenberg et al. 2017; Tang et al. 2014; Tayyrov et al. 2021; The C. elegans Sequencing Consortium 1998; Tsai et al. 2013; Wang et al. 2016, 2017; Weinstein et al. 2019; Yin et al. 2018; Young et al. 2014, 2021; Zheng et al. 2013, 2016). These included 110 parasitic species and 45 free-living species (for a full list of species, see supplementary table S3, Supplementary Material online). In cases where multiple genomes were available for a single species of helminth, the one with more predicted protein-coding genes was selected for further analysis, based on the assumption that the larger genome would be more complete. Complete BUSCO scores were obtained directly from WormBase ParaSite to assess genome completeness, with an average complete BUSCO score across our 155 species of 83% (see supplementary fig. S8, Supplementary Material online).

PfamScan (Madeira et al. 2019) (with default settings in version 1.6, against the Pfam database version 35.0) was applied to each set of predicted proteins to functionally annotate each sequence by domain architecture. Two proteins from different proteomes with an identical Pfam domain architecture are therefore considered members of the same protein family. A phylogenetic tree was constructed using abundant single-copy proteins identified by OrthoFinder, v2.5.4 with default settings (Emms and Kelly 2015, 2017, 2018, 2019). We then used this tree to display protein presence/absence data using iTOL (Letunic and Bork 2021).

### Tracking the Presence of Proteins of Interest

Query sequences for proteins of interest were identified based on known proteins from *C. elegans, H. sapiens,* and *S. cerevisiae* from the Universal Protein Knowledgebase (The UniProt Consortium et al. 2021). We additionally used queries from four other helminths, including *A. lumbricoides*, *Plectus sambesii*, *Panagrellus redivivus,* and *Romanomermis culicivorax.* These species were selected for use as they had the highest number of BLASTP hits from an initial screen of *C. elegans* proteins. Query proteins were identified for these helminths based on the protein annotations from PfamScan.

We utilized two separate methods to assess whether a protein of interest was present within each of our species. First, we downloaded the predicted protein sets for species of interest, and performed protein BLAST searches using BLASTP 2.13.0+ with an E-value threshold of 1e−3. Species for which there were “hits’ at this threshold were considered to have a gene encoding the sequence of interest, while we performed further analysis for species with no hits. For these, we also searched for our proteins of interest based on Pfam domain architectures. All the proteins of interest for this project could be represented by a single conserved Pfam domain; however, given that previous research in helminth proteomics has uncovered the presence of many unusual protein domain architectures, especially those containing multiple, unrelated domains “fuzed” together into unique protein domain architectures (Coghlan et al. 2019), we chose to additionally examine the domain architecture library we constructed to see if our proteins of interest were occurring in combination with other conserved protein families. Using our Pfam annotation files to identify protein families within the *C. elegans* query protein, we then searched for domain architectures in our protein library that matched that of the *C. elegans* protein. In many species, we identified the sequence of interest only in multidomain architectures with additional conserved domains that are not present in the query protein from *C. elegans*. If a protein was detected by either BLASTP or by its associated Pfam domain, we considered it to be present in its respective genome.

The proteins of interest to this project included enzymes involved in glycolysis, the citric acid cycle (see supplementary table S4, Supplementary Material online for abbreviations used for each protein), and the five complexes of the ETC; peroxisomal proteins; and proteins involved in defense against ROS.

We used the KEGG peroxisomal pathway (Kanehisa et al. 2021) and the Universal Protein Knowledgebase (The UniProt Consortium et al. 2021) to identify peroxisomal proteins present in *C. elegans* and used these as queries for our BLASTP searches to produce a presence/absence profile, visualized with iTOL (Letunic and Bork 2021). For a full list of peroxisomal proteins identified in *C. elegans*, along with their abbreviations and role within the peroxisome, see supplementary table S2, Supplementary Material online. Please note that the abbreviation prx is used in many eukaryotes for genes associated with peroxiredoxin; however in *C. elegans*, it is used to denote peroxin protein-coding genes.

## Supplementary Material

evad135_Supplementary_Data

## Data Availability

All data analyzed in this study are derived from genomic information publicly available from the WormBase Parasite and NCBI GenBank databases (see Materials and Methods section for details). For results from our BLASTP searches and presence/absence profiling of metabolic pathways of interest, please see the Supplementary File provided.
