# Supplementary material for: Phylogenomic Analysis of 155 Helminth Species Reveals Widespread Absence of Oxygen Metabolic Capacity
Source: Genome Biol Evol. 2023 Jul 22;15(8):evad135. doi: 10.1093/gbe/evad135 (PMC10400150; doi:10.1093/gbe/evad135)
Supplement: evad135_Supplementary_Data [file evad135_supplementary_data.zip › Collington_GBE_Sup_FINAL_r2_reduced.docx]

**Supplementary Figures for Phylogenomic analysis of 155 helminth species reveals widespread absence of oxygen metabolic capacity.**

(E. Collington, B. Lobb, N. Mazen, A. Doxey, D. M. Glerum)


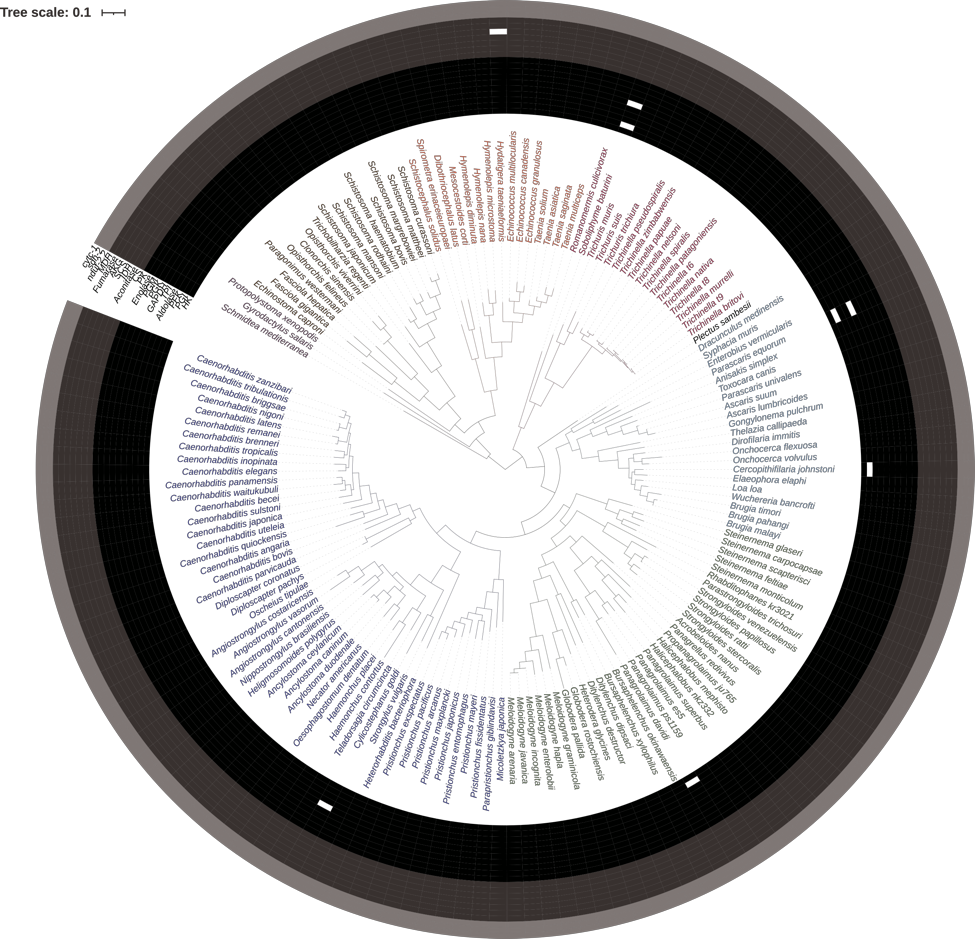


**S. Fig. 1.** The presence (coloured) and absence (white) of major aerobic respiration proteins in 155 species of helminth, visualized with iTOL. In black: glycolysis proteins, from inside to outside: HK, PGI, PFK, aldolase, TPI, GAPDH, PGK, PGM, enolase, PK; in medium grey, citric acid cycle proteins CS, aconitase, IDH, SCS, AKG, fumarase, MDH; and in light grey, *nduf-2.2* (Complex I marker), *sdh-2* (Complex II marker), and cytochrome C1 (Complex III marker). For a full list of non-abbreviated proteins, see S. Table 2.

**A.**

**
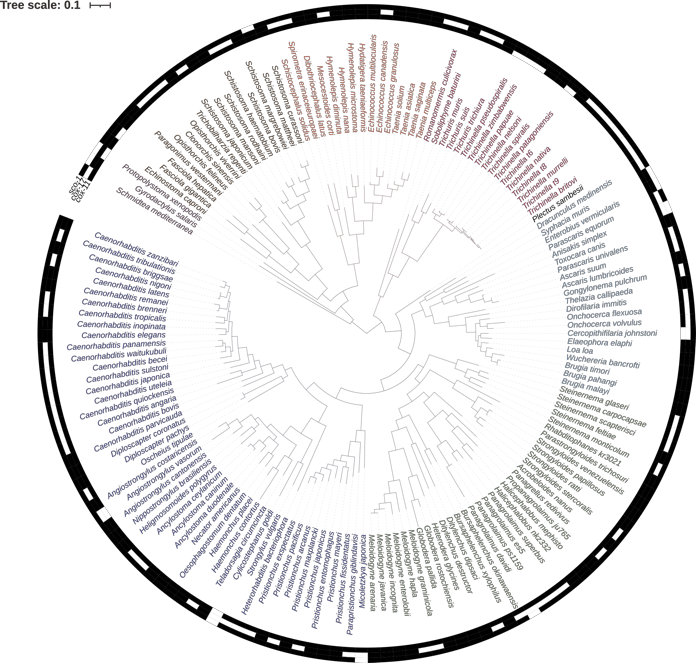
**

**B.**

**
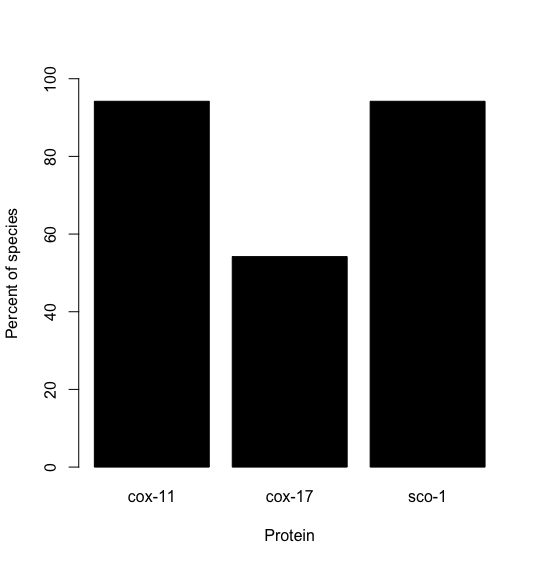
**

**S. Fig. 2. A.** Presence of essential COX assembly factors in 155 species of helminth visualized with iTOL. Essential assembly factors necessary for formation of the COX catalytic core include (from inside to outside), *cox-11*, *cox-17*, and *sco-1.* **B.** Percent of species found to contain each of the essential COX assembly factors: 94% of species investigated were found to contain a *cox-11* protein, 54% had *cox-17*, and 94% had *sco-1*.

**A**.

**
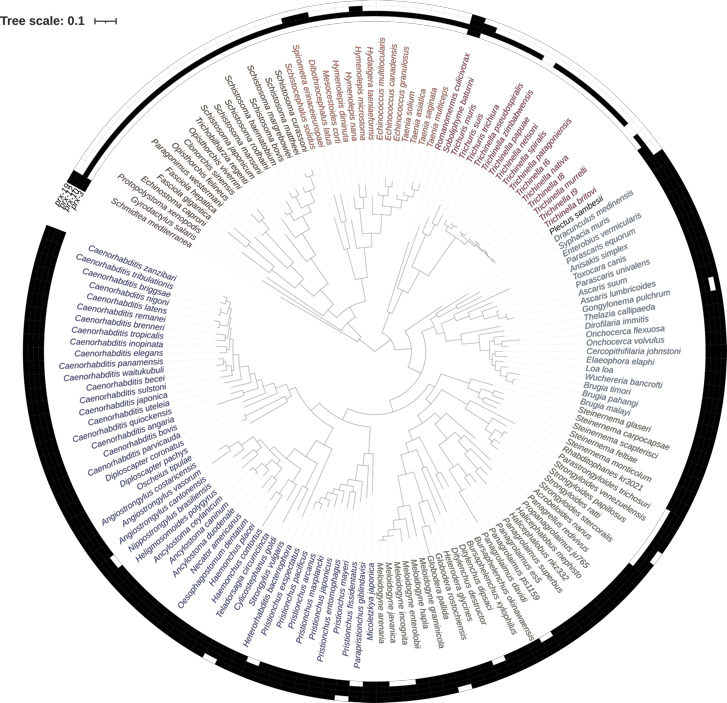
**

**B.**

**
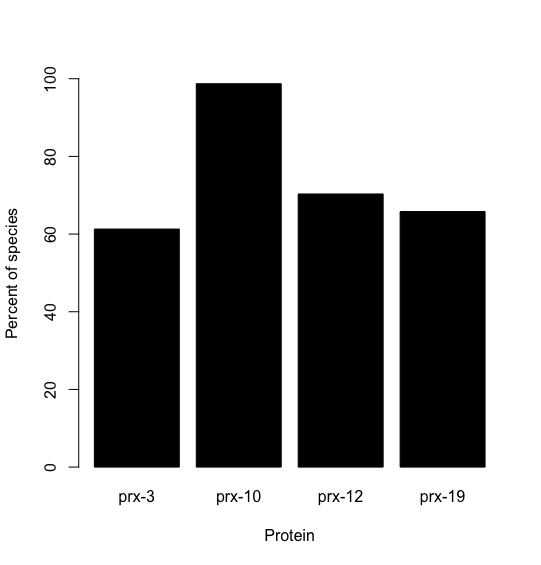
**

**S. Fig. 3. A.** Presence of essential peroxin protein-coding genes in 155 species of helminth visualized with iTOL. Genes include (from inside to outside) *prx-3*, *prx-10*, *prx-12*, *prx-19*. **B.** Percent of species identified with each of the essential peroxins: 61% of species had a *prx-3*, 98% of species contained *prx-10*, 70% had *prx-12*, and 67% had *prx-19.*


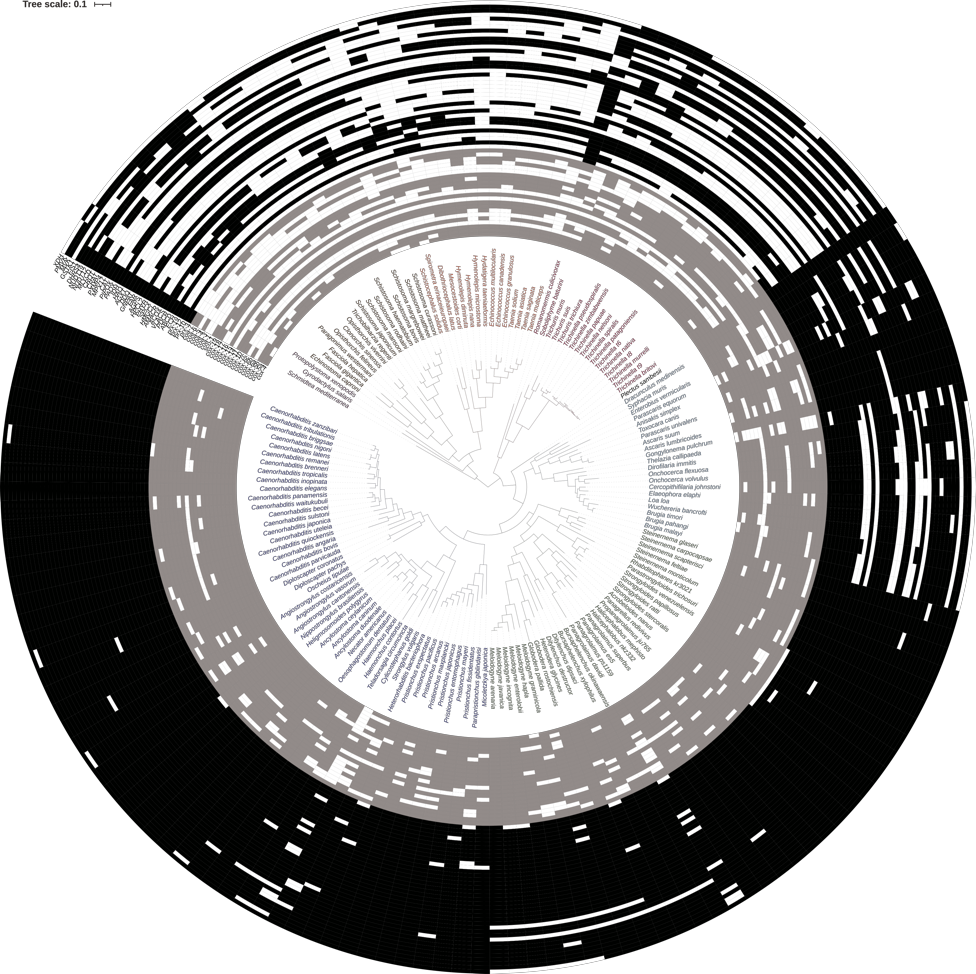


**S. Fig. 4.** Absence of peroxisomal proteins and nuclear encoded COX-related proteins in 155 species of helminth, visualized with iTOL. Heatmap showing the distribution of the absence (white) of peroxisomal proteins and COX-related proteins, including (from inside to outside): COX subunits and assembly factors in grey, *cox-4*, *cox-5a*, *cox5b*, *cox-6a*, *cox-6b*, *cox-6c*, and *cox-7c*, *cox-10*, cox*-11*, *cox-14*, *cox-15*, *cox-16*, *cox-17*, *cox-18*, *cox-19*, *sco-1, coa-1*, *coa-3*, *coa-4*, *coa-5*, *coa-6*, *coa-7*; and peroxisomal proteins in black *prx-3*, *prx-10*, *prx-12*, *prx-19*, ECH, ABCD, PDCR, VLACS, ACOX, AMACR, HMGCL, SPCX, PHYH, HPCL2, AGPS, FAR, GNPAT, DDO, AGXT, HAO, IDH, PAOX, PIPOX, CAT, MPV17, PXMP4, CRAT, CROT, MLYCD, NUDT12, NUDT19, DHRS4, FIS1, GSTK1, MVK, PMVK, and XDH. A full list of non-abbreviated proteins can be found in S. Table 3.

**S. Table 1.** Presence of genes encoding essential peroxin proteins and COX assembly factors in 155 species of helminth worm. Peroxin proteins *prx-3, prx-10, prx-12,* and *prx-19* are necessary for the formation of peroxisomes, and COX assembly factors *cox-11, cox-17*, and *sco-1* are needed for the formation of a functional COX holoenzyme. Of 155 species of helminth, 41 were found to contain all the essential proteins for the formation peroxisomes and COX, 49 were missing at least one essential COX assembly factor but retained the peroxisomal proteins, 33 species were missing at least one essential peroxin proteins but retained the assembly factors for COX, and 32 were at least one member of each set of proteins.

| Species missing at least one essential COX and one essential PEX protein | Species missing at least one essential COX protein, all essential PEX proteins present | Species missing at least one essential PEX protein, all COX proteins present | Species with all essential COX and PEX proteins |
| --- | --- | --- | --- |
| *Clonorchis sinensis* | *Ancylostoma caninum* | *Dibothriocephalus latus* | *Acrobeloides nanus* |
| *Cylicostephanus goldi* | *Ancylostoma ceylanicum* | *Ditylenchus destructor* | *Ancylostoma duodenale* |
| *Echinococcus canadensis* | *Angiostrongylus vasorum* | *Echinococcus granulosus* | *Angiostrongylus cantonensis* |
| *Fasciola gigantica* | *Anisakis simplex* | *Echinococcus multilocularis* | *Angiostrongylus costaricensis* |
| *Gongylonema pulchrum* | *Ascaris suum* | *Echinostoma caproni* | *Ascaris lumbricoides* |
| *Gyrodactylus salaris* | *Bursaphelenchus okinawaensis* | *Fasciola hepatica* | *Brugia malayi* |
| *Hymenolepis diminuta* | *Caenorhabditis angaria* | *Globodera pallida* | *Brugia pahangi* |
| *Opisthorchis felineus* | *Caenorhabditis becei* | *Hydatigera taeniaeformis* | *Brugia timori* |
| *Opisthorchis viverrini* | *Caenorhabditis bovis* | *Hymenolepis microstoma* | *Bursaphelenchus xylophilus* |
| *Panagrolaimus davidi* | *Caenorhabditis brenneri* | *Hymenolepis nana* | *Caenorhabditis briggsae* |
| *Parapristionchus giblindavisi* | *Caenorhabditis japonica* | *Meloidogyne incognita* | *Caenorhabditis elegans* |
| *Pristionchus exspectatus* | *Caenorhabditis nigoni* | *Meloidogyne javanica* | *Caenorhabditis inopinata* |
| *Protopolystoma xenopodis* | *Caenorhabditis panamensis* | *Mesocestoides corti* | *Caenorhabditis latens* |
| *Schistocephalus solidus* | *Caenorhabditis quiockensis* | *Nippostrongylus brasiliensis* | *Caenorhabditis parvicauda* |
| *Schistosoma bovis* | *Caenorhabditis sulstoni* | *Paragonimus westermani* | *Caenorhabditis remanei* |
| *Schistosoma haematobium* | *Caenorhabditis tribulationis* | *Parastrongyloides trichosuri* | *Caenorhabditis zanzibari* |
| *Schistosoma japonicum* | *Caenorhabditis tropicalis* | *Pristionchus fissidentatus* | *Diploscapter coronatus* |
| *Schistosoma mattheei* | *Caenorhabditis uteleia* | *Schistosoma curassoni* | *Diploscapter pachys* |
| *Spirometra erinaceieuropaei* | *Caenorhabditis waitukubuli* | *Schistosoma mansoni* | *Elaeophora elaphi* |
| *Strongyloides stercoralis* | *Cercopithifilari johnstoni* | *Schistosoma margrebowiei* | *Globodera rostochiensis* |
| *Taenia multiceps* | *Dirofilarial immitis* | *Schistosoma rodhaini* | *Haemonchus placei* |
| *Trichinella britovi* | *Ditylenchus dipsaci* | *Soboliphyme baturini* | *Halicephalobus mephisto* |
| *Trichinella murrelli* | *Dracunculus medinensis* | *Strongyloides papillosus* | *Halicephalobus nkz332* |
| *Trichinella nativa* | *Enterobius vermicularis* | *Strongyloides ratti* | *Heligmosomoides polygyrus* |
| *Trichinella papuae* | *Haemonchus contortus* | *Strongyloides venezuelensis* | *Heterodera glycines* |
| *Trichinella patagoniensis* | *Heterorhabditis bacteriophora* | *Taenia asiatica* | *Loa loa* |
| *Trichinella pseudospiralis* | *Meloidogyne graminicola* | *Taenia saginata* | *Meloidogyne arenaria* |
| *Trichinella t8* | *Meloidogyne hapla* | *Taenia solium* | *Meloidogyne enterolobii* |
| *Trichinella t9* | *Micoletzkya japonica* | *Trichinella nelsoni* | *Necator americanus* |
| *Trichinella zimbabwensis* | *Onchocerca flexuosa* | *Trichinella spiralis* | *Oesophagostomum dentatum* |
| *Trichuris muris* | *Onchocerca volvulus* | *Trichinella t6* | *Pristionchus entomophagus* |
| *Trichuris suis* | *Oscheius tipulae* | *Trichobilharzia regenti* | *Pristionchus maxplancki* |
|  | *Panagrellus redivivus* | *Trichuris trichiura* | *Pristionchus mayeri* |
|  | *Panagrolaimus es5* |  | *Pristionchus pacificus* |
|  | *Panagrolaimus ps1159* |  | *Propanagrolaimus ju765* |
|  | *Panagrolaimus superbus* |  | *Rhabditophanes kr3021* |
|  | *Parascaris equorum* |  | *Steinernema feltiae* |
|  | *Parascaris univalens* |  | *Teladorsagia circumcincta* |
|  | *Plectus sambesii* |  | *Thelazia callipaeda* |
|  | *Pristionchus arcanus* |  | *Toxocara canis* |
|  | *Pristionchus japonicus* |  | *Wuchereria bancrofti* |
|  | *Romanomermis culicivorax* |  |  |
|  | *Schmidtea mediterranea* |  |  |
|  | *Steinernema carpocapsae* |  |  |
|  | *Steinernema glaseri* |  |  |
|  | *Steinernema monticolum* |  |  |
|  | *Steinernema scapterisci* |  |  |
|  | *Strongylus vulgaris* |  |  |
|  | *Syphacia muris* |  |  |


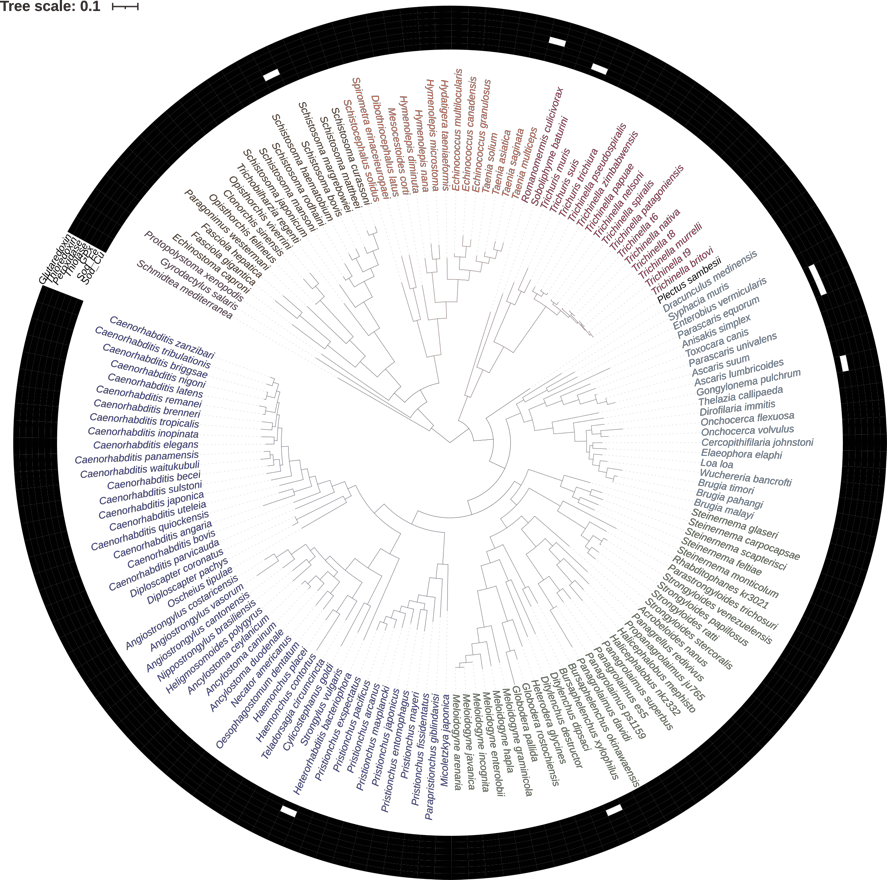


**S. Fig. 5.** Antioxidant protein-coding genes in 155 species of helminth, visualized with iTOL. Genes include (from inside to outside), superoxide dismutases Sod_Cu and Sod_Fe, glutathione peroxidase, thiolase, peroxidase, thioredoxin, and glutaredoxin.

**
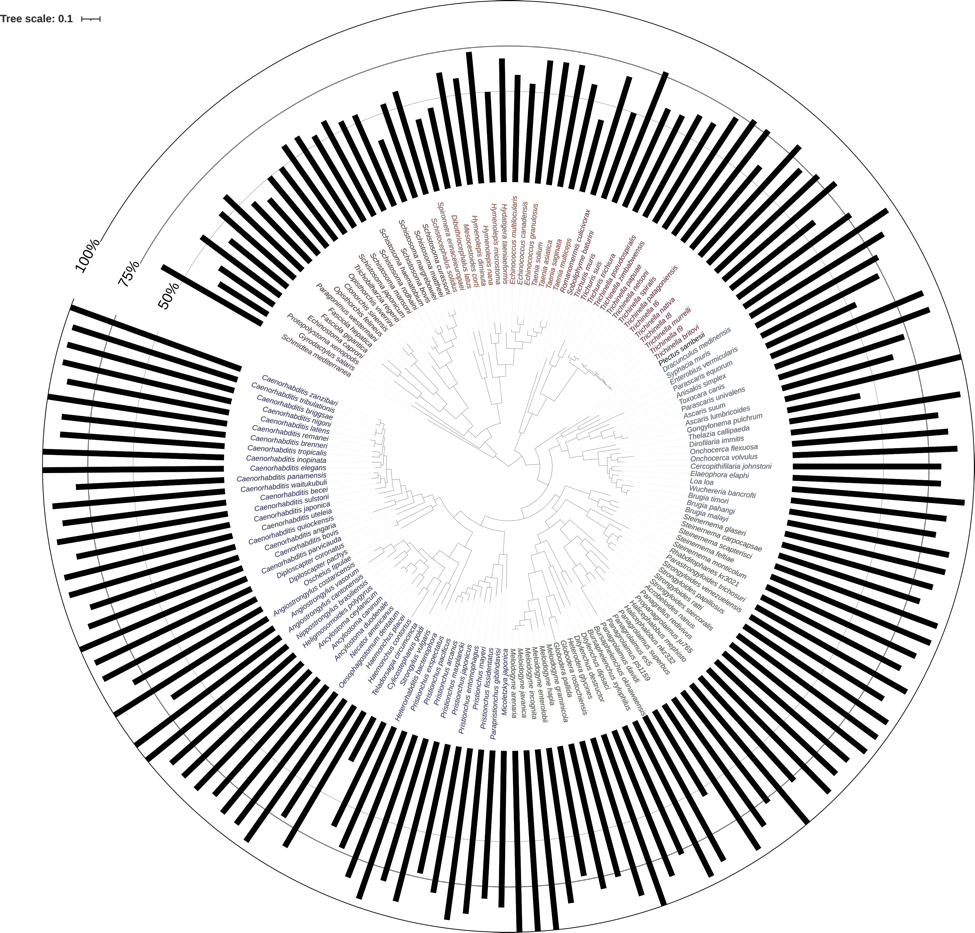
**

**S. Fig. 6.** The percentage of COX-related proteins present in each species is indicated as a bar graph on the phylogenetic tree. The outer line in black represents 100%, the grey line 75%, and the innermost red line indicates 50%.


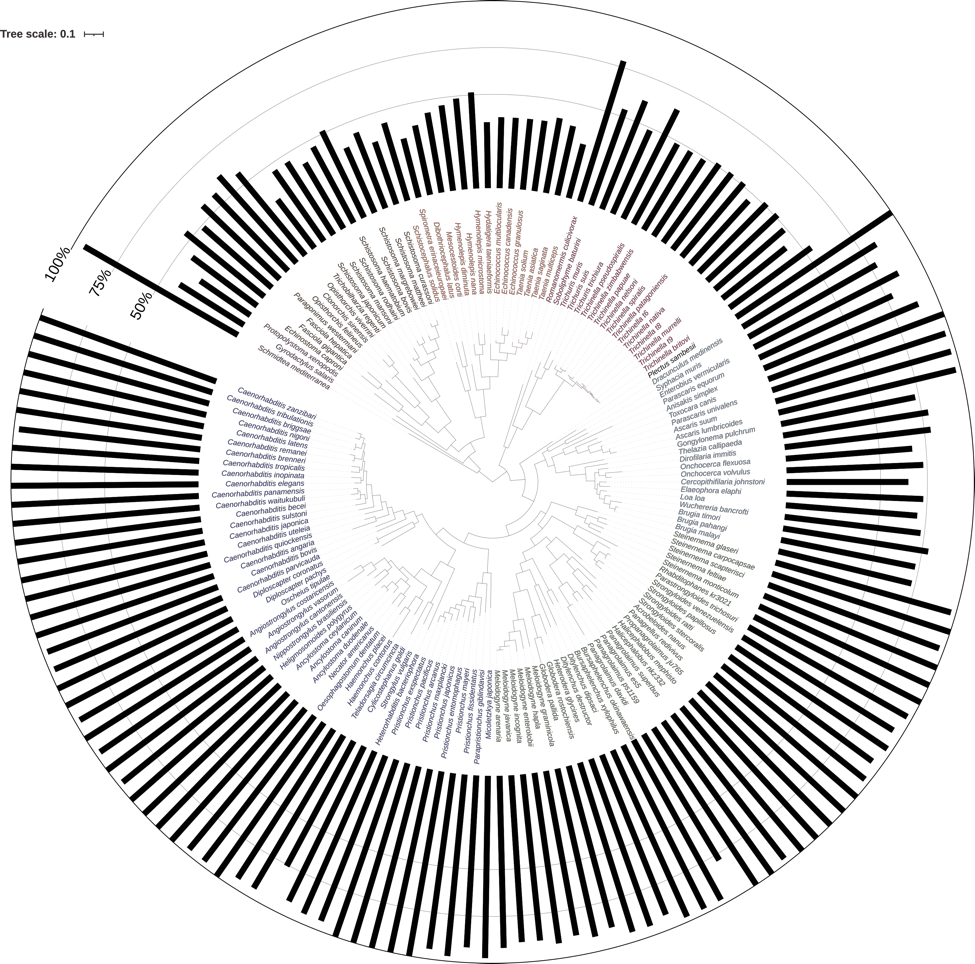


**S. Fig. 7**. The percentage of peroxisomal proteins of interest present in species on the phylogenetic tree, indicated as a bar graph. The outer line in black represents 100%, the grey line 75%, and the innermost red line indicates 50%.

**S. Table 2.** Peroxisomal proteins in *C. elegans*. Proteins involved in different peroxisomal pathways from *C. elegans* were identified and their sequences used to determine the presence of these proteins in other species of helminth. The abbreviation *prx* is used in many eukaryotes for genes associated with peroxiredoxin, however in *C. elegans* it is used to denote peroxin proteins.

| Pathway | Protein | Abbreviation |
| --- | --- | --- |
| Peroxins | Pex3 | *prx-3* |
|  | Pex10 | *prx-10* |
|  | Pex12 | *prx-12* |
|  | Pex19 | *prx-19* |
| Unsaturated fatty acid beta oxidation | Delta3 5-delta2 4-dienoyl-coa | ECH |
|  | ATP-binding cassette, subfamily | ABCD |
|  | 2,4-dienoyl-CoA reductase | PDCR |
|  | Solute carrier family 27 member | VLACS |
| Beta oxidation | Acyl-CoA oxidase | ACOX |
|  | Alpha-methylacyl-CoA racemase | AMACR |
|  | Hydroxymethylglutaryl-CoA lyase | HMGCL |
|  | Sterol carrier protein | SPCX |
| Alpha oxidation | Phytanoyl-CoA hydroxylase | PHYH |
|  | 2-hydroxyacyl-CoA lyase | HPCL2 |
| Etherphospholipid biosynthesis | Alkyldihydroxyacetonephosphate synthase | AGPS |
|  | Alcohol-forming fatty acyl-CoA reductase | FAR |
|  | Glyceronephosphate O-acyltransferase | GNPAT |
| Amino acid metabolism | D-aspartate oxidase | DDO |
|  | Alanine-glyoxylate transaminase | AGXT |
|  | (S)-2-hydroxy-acid oxidase | HAO |
|  | Isocitrate dehydrogenase | IDH |
|  | N1-acetylpolyamine oxidase | PAOX |
|  | Sarcosine oxidase | PIPOX |
| Reactive oxygen species metabolism | Catalase | CAT |
|  | Protein Mpv17 | MPV17 |
|  | Peroxisomal membrane protein 2 | PXMP4 |
| Other oxidation | Carnitine O-acetyltransferase | CRAT |
|  | Carnitine O-octanoyltransferase | CROT |
|  | Malonyl-CoA decarboxylase | MLYCD |
|  | NAD+ diphosphatase | NUDT12 |
|  | Nucleoside diphosphate-linked moiety X motif | NUDT19 |
| Retinol metabolism | Dehydrogenase/reductase SDR family member 4 | DHRS4 |
| Organelle fission | Peroxisome fission protein | FIS1 |
| Glutathione metabolism | Glutathione S-transferase kappa 1 | GSTK1 |
| Sterol precursor biosynthesis | Mevalonate kinase | MVK |
|  | Phosphomevalonate kinase | PMVK |
| Purine metabolism | Xanthine dehydrogenase | XDH |


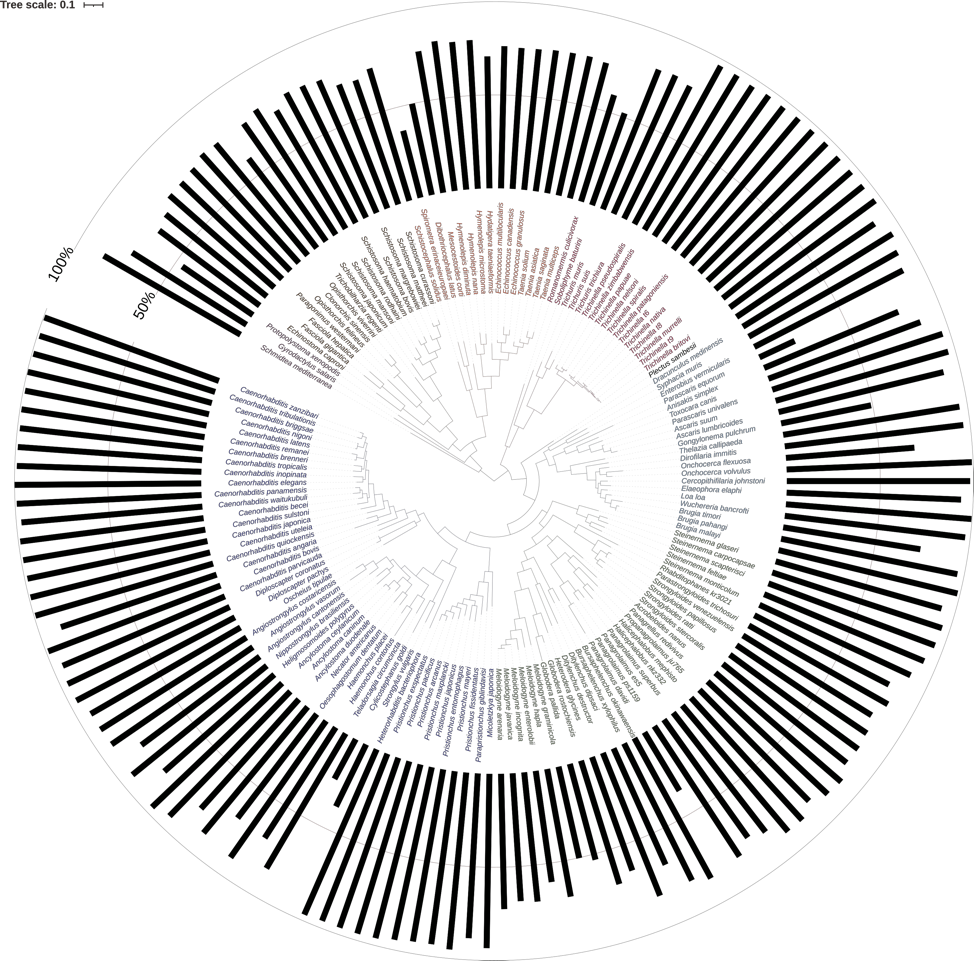


**S. Fig. 8.** Complete BUSCO scores for 155 species of helminth**.** Complete BUSCO scores for species were obtained directly from WormBase ParaSite and displayed as percentage bars using iTOL. The 100% and 50% marks are shown by the black and red lines respectively. Scores ranged from 20.8% (*Parascaris equorum*) to 100% (*Caenorhabditis elegans*).

**S. Table 3.** Genome assemblies of 155 species of helminth

| Species | Type | Genome assembly ID | Host |
| --- | --- | --- | --- |
| *Ancylostoma caninum* | Parasitic | A_caninum_9.3.2.ec.cg.pg | Dogs |
| *Ancylostoma ceylanicum* | Parasitic | GCA_000688135.1 | Humans, hamsters |
| *Ancylostoma duodenale* | Parasitic | GCA_000816745.1 | Humans |
| *Angiostrongylus cantonensis* | Parasitic | GCA_000950995.1 | Rats, humans, snails, slugs, crab, shrimp |
| *Angiostrongylus costaricensis* | Parasitic | GCA_900624975.1 | Rats, humans, snails, slugs, crab, shrimp |
| *Angiostrongylus vasorum* | Parasitic | GCA_018806985.1 | Dogs, foxes |
| *Anisakis simplex* | Parasitic | GCA_900617985.1 | Humans, marine mammals, fish |
| *Ascaris lumbricoides* | Parasitic | GCA_000951055.1 | Humans |
| *Ascaris suum* | Parasitic | GCA_000187025.3 | Pigs, humans |
| *Brugia malayi* | Parasitic | GCA_000002995.5 | Mosquitoes, humans |
| *Brugia pahangi* | Parasitic | GCA_900618355.1 | Mosquitoes, cats, dogs |
| *Brugia timori* | Parasitic | GCA_900618025.1 | Mosquitoes, humans |
| *Bursaphelenchus okinawaensis* | Parasitic | GCA_904066225.2 | Pine trees |
| *Bursaphelenchus xylophilus* | Parasitic | GCA_000231135.1 | Pine trees |
| *Cercopithifilaria johnstoni* | Parasitic | PRJEB47283 | Murids, marsupials |
| *Clonorchis sinensis* | Parasitic | GCA_003604175.1 | Human |
| *Cylicostephanus goldi* | Parasitic | GCA_900617965.1 | Horses |
| *Dibothriocephalus latus* | Parasitic | GCA_900617775.1 | Fish, humans |
| *Dirofilaria immitis* | Parasitic | nDi.2.2, University of Edinburgh | Dogs, cats, mosquitoes |
| *Ditylenchus destructor* | Parasitic | GCA_001579705.1 | Potatoes |
| *Ditylenchus dipsaci* | Parasitic | GCA_004194705.1 | Onion, garlic |
| *Dracunculus medinensis* | Parasitic | GCA_000946415.1 | Humans |
| *Echinococcus canadensis* | Parasitic | GCA_900004735.1 | Dogs |
| *Echinococcus granulosus* | Parasitic | GCA_000524195.1 | Dogs |
| *Echinococcus multilocularis* | Parasitic | GCA_000469725.3 | Dogs, humans |
| *Echinostoma caproni* | Parasitic | GCA_900618425.1 | Humans |
| *Elaeophora elaphi* | Parasitic | GCA_000499685.1 | Deer, sheep |
| *Enterobius vermicularis* | Parasitic | GCA_900576705.1 | Humans |
| *Fasciola gigantica* | Parasitic | GCA_006461475.1 | Ruminants, snails, humans |
| *Fasciola hepatica* | Parasitic | PRJNA179522 | Humans, ruminants |
| *Globodera pallida* | Parasitic | GCA_000724045.1 | Potatoes |
| *Globodera rostochiensis* | Parasitic | GCA_900079975.1 | Potatoes, tomatoes |
| *Gongylonema pulchrum* | Parasitic | GCA_900617915.1 | Humans, insects |
| *Gyrodactylus salaris* | Parasitic | GCA_000715275.1 | Freshwater fish |
| *Haemonchus contortus* | Parasitic | Hco_v4_coding_submitted, University of Melbourne | Ruminants |
| *Haemonchus placei* | Parasitic | GCA_900617895.1 | Ruminants |
| *Heligmosomoides polygyrus* | Parasitic | GCA_900618505.1 | Rodents |
| *Heterodera glycines* | Parasitic | GCA_004148225.1 | Soybeans |
| *Heterorhabditis bacteriophora* | Parasitic | GCA_000223415.1 | Insects |
| *Hydatigera taeniaeformis* | Parasitic | GCA_900622495.1 | Cats, rodents |
| *Hymenolepis diminuta* | Parasitic | GCA_902177915.1 | Rodents, insects |
| *Hymenolepis microstoma* | Parasitic | GCA_000469805.3 | Rodents |
| *Hymenolepis nana* | Parasitic | GCA_900617975.1 | Rodents, humans |
| *Loa loa* | Parasitic | GCA_000183805.2 | Humans |
| *Meloidogyne arenaria* | Parasitic | GCA_900003985.1 | Plants |
| *Meloidogyne enterolobii* | Parasitic | GCA_003693675.1 | Plants |
| *Meloidogyne graminicola* | Parasitic | GCA_002778205.1 | Rice |
| *Meloidogyne hapla* | Parasitic | GCA_000172435.1 | Plants |
| *Meloidogyne incognita* | Parasitic | GCA_900182535.1 | Plants |
| *Meloidogyne javanica* | Parasitic | GCA_900003945.1 | Plants |
| *Mesocestoides corti* | Parasitic | GCA_900604375.1 | Rodents, cats, dogs, birds |
| *Necator americanus* | Parasitic | GCA_000507365.1 | Humans |
| *Nippostrongylus brasiliensis* | Parasitic | GCA_900618405.1 | Rats |
| *Oesophagostomum dentatum* | Parasitic | GCA_000797555.1 | Pigs |
| *Onchocerca flexuosa* | Parasitic | GCA_900618345.1 | Deer |
| *Onchocerca volvulus* | Parasitic | GCA_000499405.2 | Humans, blackflies |
| *Opisthorchis felineus* | Parasitic | GCA_004794785.1 | Humans, freshwater fish |
| *Opisthorchis viverrini* | Parasitic | GCF_000715545.1 | Humans, freshwater fish |
| *Paragonimus westermani* | Parasitic | GCA_008508345.1 | Humans, snails, crustaceans |
| *Parascaris equorum* | Parasitic | PRJEB514 | Horses |
| *Parascaris univalens* | Parasitic | GCA_002259205.1 | Horses |
| *Parastrongyloides trichosuri* | Parasitic | GCA_000941615.1 | Possums |
| *Protopolystoma xenopodis* | Parasitic | CAAALY010000000 | Xenopus frogs |
| *Romanomermis culicivorax* | Parasitic | GCA_001039655.1 | Mosquitoes |
| *Schistocephalus solidus* | Parasitic | GCA_900618435.1 | Fish, birds, rodents |
| *Schistosoma bovis* | Parasitic | GCA_003958945.1 | Sheep, cattle, goats |
| *Schistosoma curassoni* | Parasitic | GCA_900618015.1 | Sheep, cattle, goats |
| *Schistosoma haematobium* | Parasitic | GCF_000699445.1 | Snails, humans |
| *Schistosoma japonicum* | Parasitic | GCA_006368765.1 | Snails, humans |
| *Schistosoma mansoni* | Parasitic | GCA_000237925.3 | Snails, humans |
| *Schistosoma margrebowiei* | Parasitic | GCA_900618395.1 | Snails, mammals |
| *Schistosoma mattheei* | Parasitic | GCA_900617995.1 | Bovid ruminants, snails, humans |
| *Schistosoma rodhaini* | Parasitic | GCA_000951475.1 | Snails, rodents |
| *Soboliphyme baturini* | Parasitic | GCA_900618415.1 | Holarctic mustelids |
| *Spirometra erinaceieuropaei* | Parasitic | GCA_000951995.1 | Humans, domestic animals, copepods, frogs, snakes |
| *Steinernema carpocapsae* | Parasitic | GCA_000757645.3 | Insects |
| *Steinernema feltiae* | Parasitic | S_felt_v1_submitted, California Institute of Technology | Insects |
| *Steinernema glaseri* | Parasitic | GCA_000757755.1 | Insects |
| *Steinernema monticolum* | Parasitic | S_monti_v1_submitted, California Institute of Technology | Insects |
| *Steinernema scapterisci* | Parasitic | S_scapt_v1_submitted, California Institute of Technology | Insects |
| *Strongyloides papillosus* | Parasitic | GCA_000936265.1 | Ruminants, pigs, rodents |
| *Strongyloides ratti* | Parasitic | GCA_001040885.1 | Rats |
| *Strongyloides stercoralis* | Parasitic | GCA_000947215.1 | Humans |
| *Strongyloides venezuelensis* | Parasitic | GCA_001028725.1 | Mice |
| *Strongylus vulgaris* | Parasitic | GCA_900624965.1 | Horses, donkeys |
| *Syphacia muris* | Parasitic | GCA_000939275.1 | Rats |
| *Taenia asiatica* | Parasitic | GCA_900618005.1 | Humans, pigs |
| *Taenia multiceps* | Parasitic | GCA_001923025.3 | Dogs, wolves, foxes, ruminants, humans |
| *Taenia saginata* | Parasitic | GCA_001693075.2 | Cattle, humans |
| *Taenia solium* | Parasitic | Tsolium_Mexico_v1, National University of Mexico | Pigs, humans |
| *Teladorsagia circumcincta* | Parasitic | GCA_002352805.1 | Sheep |
| *Thelazia callipaeda* | Parasitic | GCA_900618365.1 | Mammals |
| *Toxocara canis* | Parasitic | GCA_900622545.1 | Dogs, humans |
| *Trichinella britovi* | Parasitic | GCA_001447585.1 | Carnivores, pigs, horses |
| *Trichinella murrelli* | Parasitic | GCA_001447425.1 | Bears, raccoons, foxes, bob cats, coyotes |
| *Trichinella nativa* | Parasitic | GCA_001447565.1 | Mammals, birds |
| *Trichinella nelsoni* | Parasitic | GCA_001447455.1 | Carnivores, scavengers |
| *Trichinella papuae* | Parasitic | GCA_001447755.1 | Pigs, crocodiles |
| *Trichinella patagoniensis* | Parasitic | GCA_001447655.1 | Carnivorous mammals |
| *Trichinella pseudospiralis* | Parasitic | GCA_001447675.1 | Mammals, birds |
| *Trichinella spiralis* | Parasitic | GCA_001447595.1 | Pigs, humans |
| *Trichinella sp. t6* | Parasitic | GCA_001447435.1 | Mammals, birds |
| *Trichinella sp. t8* | Parasitic | GCA_001447745.1 | Lions, hyenas |
| *Trichinella sp. t9* | Parasitic | GCA_001447505.1 | Carnivores |
| *Trichinella zimbabwensis* | Parasitic | GCA_001447665.1 | Crocodiles, monitor lizards |
| *Trichobilharzia regenti* | Parasitic | GCA_900618515.1 | Birds, snails, humans |
| *Trichuris muris* | Parasitic | GCA_000612645.2 | Mice |
| *Trichuris suis* | Parasitic | GCA_000701005.1 | Pigs |
| *Trichuris trichiura* | Parasitic | GCA_000613005.1 | Humans |
| *Wuchereria bancrofti* | Parasitic | GCA_000180755.1 | Humans |
| *Acrobeloides nanus* | Free-living | GCA_900406225.1 |  |
| *Caenorhabditis angaria* | Free-living | PRJNA51225, California Institute of Technology |  |
| *Caenorhabditis becei* | Free-living | GCA_900536315.3 |  |
| *Caenorhabditis bovis* | Free-living | GCA_902829315.1 |  |
| *Caenorhabditis brenneri* | Free-living | GCA_000143925.2 |  |
| *Caenorhabditis briggsae* | Free-living | GCA_000004555.3 |  |
| *Caenorhabditis elegans* | Free-living | GCA_000002985.3 |  |
| *Caenorhabditis inopinata* | Free-living | GCA_003052745.1 |  |
| *Caenorhabditis japonica* | Free-living | GCA_000147155.1 |  |
| *Caenorhabditis latens* | Free-living | GCA_002259235.1 |  |
| *Caenorhabditis nigoni* | Free-living | GCA_002742825.1 |  |
| *Caenorhabditis panamensis* | Free-living | GCA_900536275.1 |  |
| *Caenorhabditis parvicauda* | Free-living | GCA_900536235.3 |  |
| *Caenorhabditis quiockensis* | Free-living | GCA_900536415.3 |  |
| *Caenorhabditis remanei* | Free-living | GCA_000149515.1 |  |
| *Caenorhabditis sulstoni* | Free-living | GCA_900536325.3 |  |
| *Caenorhabditis tribulationis* | Free-living | GCA_900536305.3 |  |
| *Caenorhabditis tropicalis* | Free-living | GCA_000186765.1 |  |
| *Caenorhabditis uteleia* | Free-living | GCA_900536295.3 |  |
| *Caenorhabditis waitukubuli* | Free-living | GCA_900536345.3 |  |
| *Caenorabditis zanzibari* | Free-living | GCA_900536285.3 |  |
| *Diploscapter coronatus* | Free-living | GCA_002207785.1 |  |
| *Diploscapter pachys* | Free-living | GCA_002287525.1 |  |
| *Halicephalobus mephisto* | Free-living | SWDT00000000 |  |
| *Halicephalobus nkz332* | Free-living | GCA_009761265.1 |  |
| *Micoletzkya japonica* | Free-living | GCA_900490955.1 |  |
| *Oscheius tipulae* | Free-living | GCA_900184235.1 |  |
| *Panagrellus redivivus* | Free-living | GCA_000341325.1 |  |
| *Panagrolaimus davidi* | Free-living | GCA_901779475.1 |  |
| *Panagrolaimus es5* | Free-living | GCA_901766855.1 |  |
| *Panagrolaimus ps1159* | Free-living | GCA_901765195.1 |  |
| *Panagrolaimus superbus* | Free-living | GCA_901766145.1 |  |
| *Parapristionchus giblindavisi* | Free-living | GCA_900491355.1 |  |
| *Plectus sambesii* | Free-living | GCA_002796945.1 |  |
| *Pristionchus arcanus* | Free-living | GCA_900490705.1 |  |
| *Pristionchus entomophagus* | Free-living | GCA_900490825.1 |  |
| *Pristionchus exspectatus* | Free-living | GCA_900380275.1 |  |
| *Pristionchus fissidentatus* | Free-living | GCA_900490895.1 |  |
| *Pristionchus japonicus* | Free-living | GCA_900490845.1 |  |
| *Pristionchus maxplancki* | Free-living | GCA_900490775.1 |  |
| *Pristionchus mayeri* | Free-living | GCA_900490875.1 |  |
| *Pristionchus pacificus* | Free-living | GCA_000180635.3 |  |
| *Propanagrolaimus ju765* | Free-living | GCA_901765185.1 |  |
| *Rhabditophanes kr3021* | Free-living | GCA_000944355.1 |  |
| *Schmidtea mediterranea* | Free-living | GCA_002600895.1 |  |

**S. Table 4.** Glycolytic and citric acid cycle proteins. Sequences from major glycolytic and citric acid cycle enzymes from *C. elegans* were used to search for these proteins in our helminth species of interest. Abbreviations are shown where applicable.

| Pathway | Enzyme | Abbreviation |
| --- | --- | --- |
| Glycolysis | Hexokinase | HK |
|  | Phosphoglucose isomerase | PGI |
|  | Phosphofructokinase | PFK |
|  | Fructose bisphosphate aldolase | Aldolase |
|  | Triosephosphate isomerase | TPI |
|  | Glyceraldehyde phosphate dehydrogenase | GAPDH |
|  | Phosphoglycerate kinase | PGK |
|  | Phosphoglycerate mutase | PGM |
|  | Enolase |  |
|  | Pyruvate kinase | PK |
| Citric acid cycle | Citrate synthase | CS |
|  | Aconitase |  |
|  | Isocitrate dehydrogenase | IDH |
|  | Succinyl-CoA synthetase | SCS |
|  | Alpha ketoglutarate | AKG |
|  | Fumarase |  |
|  | Malate dehydrogenase | MDH |
